# Astaxanthin, Compared to Other Carotenoids, Increases the Efficacy of Methotrexate in Rat Adjuvant Arthritis

**DOI:** 10.3390/ijms25168710

**Published:** 2024-08-09

**Authors:** Katarína Pružinská, Martin Chrastina, Sasan Khademnematolahi, Veronika Vyletelová, Lívia Gajdošová, Lucia Pastvová, František Dráfi, Silvester Poništ, Ľudmila Pašková, Jarmila Kucharská, Zuzana Sumbalová, Jana Muchová, Silvia Martiniaková, Katarína Bauerová

**Affiliations:** 1 Jessenius Faculty of Medicine in Martin, Comenius University in Bratislava, Malá Hora 10701/4A, 036 01 Martin, Slovakia; katarina.pruzinska@savba.sk; 2 Institute of Experimental Pharmacology and Toxicology, Centre of Experimental Medicine SAS, 841 04 Bratislava, Slovakia; martin.chrastina@savba.sk (M.C.); sasan.khademnematolahi@savba.sk (S.K.); frantisek.drafi@savba.sk (F.D.); silvester.ponist@savba.sk (S.P.); 3 Faculty of Natural Sciences, Comenius University in Bratislava, Ilkovičova 6, 842 15 Bratislava, Slovakia; 4 Faculty of Pharmacy, Comenius University in Bratislava, Odbojárov 10, 832 32 Bratislava, Slovakia; vyletelova@fpharm.uniba.sk (V.V.); paskova@fpharm.uniba.sk (Ľ.P.); 5 Institute of Medical Chemistry, Biochemistry and Clinical Biochemistry, Faculty of Medicine, Comenius University, Sasinkova 2, 813 72 Bratislava, Slovakia; livia.gajdosova@fmed.uniba.sk (L.G.); cadrova5@uniba.sk (L.P.); zuzana.sumbalova@fmed.uniba.sk (Z.S.); jana.muchova@fmed.uniba.sk (J.M.); 6 Pharmacobiochemical Laboratory of Third Department of Internal Medicine, Faculty of Medicine, Comenius University in Bratislava, Špitálska 24, 813 72 Bratislava, Slovakia; jarmila.kucharska@fmed.uniba.sk; 7 Department of Food Technology, Institute of Food Science and Nutrition, Faculty of Chemical and Food Technology, Slovak University of Technology in Bratislava, Radlinského 9, 812 37 Bratislava, Slovakia; silvia.martiniakova@stuba.sk

**Keywords:** astaxanthin, carotenoids, adjuvant arthritis, methotrexate, inflammation, oxidative stress, beta-cryptoxanthin, beta-carotene

## Abstract

This in vivo study performed in rat adjuvant arthritis aims to advance the understanding of astaxanthin’s therapeutic properties for the possible treatment of rheumatoid arthritis (RA) in monotherapy and along with the standard RA treatment, methotrexate (MTX), in combination therapy. The main goal was to elucidate astaxanthin’s full therapeutic potential, evaluate its dose dependency, and compare its effects in monotherapy with other carotenoids such as β-carotene and β-cryptoxanthin (KXAN). Moreover, potential differences in therapeutic activity caused by using different sources of astaxanthin, synthetic (ASYN) versus isolated from *Blakeslea trispora* (ASTAP), were evaluated using one-way ANOVA (Tukey-Kramer post hoc test). KXAN was the most effective in reducing plasma MMP-9 levels in monotherapy, significantly better than MTX, and in reducing hind paw swelling. The differences in the action of ASTAP and ASYN have been observed across various biometric, anti-inflammatory, and antioxidative parameters. In combined therapy with MTX, the ASYN + MTX combination proved to be better. These findings, especially the significant anti-arthritic effect of KXAN and ASYN + MTX, could be the basis for further preclinical studies.

## 1. Introduction

Astaxanthin, a naturally occurring carotenoid pigment found predominantly in marine organisms, e.g., shrimp [1], salmon [2], and algae species such as *Haematococcus pluvialis* [3,4,5], has garnered considerable attention in recent years due to its potent antioxidant properties [6], primarily via the nuclear factor erythroid 2–related factor 2 (Nrf2)/Kelch-like ECH-associated protein 1 (Keap1) pathway [7] and anti-inflammatory properties [8]. As a member of the xanthophyll family [9], astaxanthin exhibits unique molecular characteristics, including its ability to quench singlet oxygen [10]. Of the many naturally occurring carotenoids, astaxanthin is considered one of the best due to its ability to protect cells, lipids, and membrane lipoproteins from oxidative damage [3].

The burgeoning interest in astaxanthin stems from its potential therapeutic applications across various health conditions, mainly cardiovascular diseases [11], neurodegenerative disorders [12,13,14], skin aging [15], and ocular ailments [16].

In vitro studies have provided promising insights into its mechanisms of action, suggesting its role in modulating oxidative stress (OS) [17], inflammation [18], and cellular signaling pathways implicated in disease pathogenesis [19]. Astaxanthin has exhibited remarkable inhibitory effects on lipid peroxidation, surpassing vitamin E (α-tocopherol) by 100–500 times [20]. Moreover, it demonstrates several-fold higher antioxidant activity against free radicals compared to synthetic antioxidants [21], vitamin E, and β-carotene [22]. One of the ways astaxanthin reduces inflammatory markers is by blocking the nuclear factor-kB (NF-κB)-dependent signaling pathway, thus inhibiting the gene expression of inflammatory mediators such as IL-1β, IL-6, and tumor necrosis factor α (TNF-α) [23].

Astaxanthin has been studied in multiple animal models of several pathologies, such as rat hepatotoxicity [24], models mimicking cardiovascular disease in hypercholesterolemic rabbits [25], ethanol-induced gastric damage [26], colorectal carcinogenesis in obese mice [27], and nonalcoholic steatohepatitis [28].

Other carotenoids, such as β-carotene and β-cryptoxanthin, also exhibit interesting properties.

β-Carotene showed an inhibitory effect on the expression of pro-inflammatory mediators such as nitric oxide (NO), prostaglandin E2 (PGE2), inducible nitric oxide synthase (iNOS), cyclooxygenase-2 (COX-2), TNF-α, and IL-1β by acting as an inhibitor of NF-κB activation [29]. β-cryptoxanthin, though less studied, has been shown to decrease the expression of inflammatory cytokines (IL-1β, TNF-α, and IL-6) and matrix metalloproteinase 13 (MMP-13) in the primary chondrocytes of mice, leading to amelioration of osteoarthritis development [30]. 

In our study, two in vivo experiments (pilot and pivotal) were set up to evaluate the therapeutic potential of astaxanthin using adjuvant arthritis (AA), a well-established animal model of rheumatoid arthritis (RA) that reflects RA in humans [31,32,33]. The aim was to evaluate its systemic effects on critical biomarkers associated with OS and inflammation (in plasma and tissues—spleen and liver) by administering astaxanthin through oral supplementation for 28 days. 

Through our established experimental design [34,35,36], careful parameter analysis, and data interpretation experience [37], this in vivo study aimed to advance the understanding of astaxanthin’s therapeutic properties and pave the way for its possible clinical investigation along with the standard treatment with methotrexate (MTX) in combination therapy. Our study will contribute to the armamentarium currently available about the biological activities of astaxanthin, primarily by comparing the anti-rheumatic effects of natural and synthetic astaxanthin, monitoring the changes in the expression of liver heme oxygenase-1 (OH-1), platelet-activating factor-acetylhydrolase (PAF-AH), Trolox equivalent antioxidant capacity (TEAC), and liver lipoperoxide levels (LPx), as well as the activities of glutathione peroxidase (GPx), catalase (CAT), and erythrocyte superoxide dismutase (SOD). This article presents unique results about the anti-rheumatic activity of β-cryptoxanthin, including changes in the volume of the hind paw, the arthrogram, and the plasmatic levels of matrix metalloproteinase-9 (MMP-9).

The goal was to elucidate the full therapeutic potential of astaxanthin, which might improve the prevention and management of RA, and to compare its effect in monotherapy with other carotenoids, such as β-carotene and β-cryptoxanthin. 

Moreover, potential differences in therapeutic activity between synthetic and Blakeslea trispora-derived astaxanthin were also evaluated.

## 2. Results

### 2.1. Results from the Pilot Experiment

The pilot experiment, a dose-finding study based on astaxanthin monotherapy, set the trajectory for the next pivotal investigation. In both studies, we studied the pathophysiology of AA and the changes in inflammatory parameters and OS achieved with astaxanthin treatment.

#### 2.1.1. Biometric Results from the Pilot Experiment 

##### The Change in the Weight of the Animals during the Pilot Experiment

**Figure 1:**
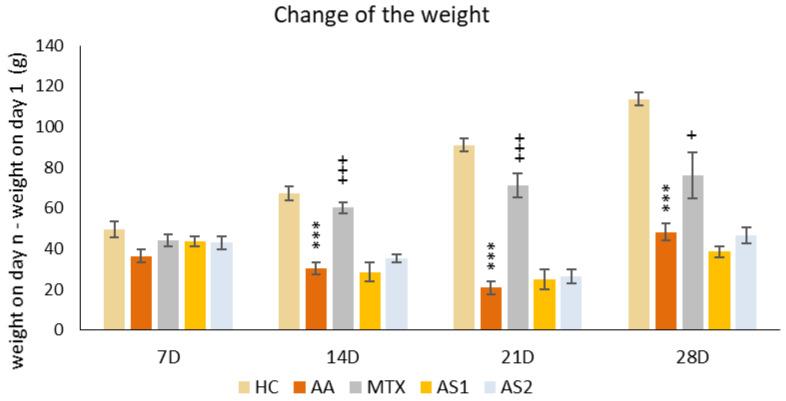
The presented data show the change in animal body weight measured on days 7, 14, 21, and 28 of the pilot experiment. The experimental animals were divided as follows: HC—healthy control group; AA—group of untreated rats with adjuvant arthritis (AA); MTX—rats with AA treated with methotrexate at a dose of 0.3 mg/kg twice weekly (MTX); AS1—group of rats with AA receiving astaxanthin of natural origin in the oral dose of 1 mg/kg daily; AS2—group of rats with AA receiving astaxanthin of natural origin in the oral dose of 5 mg/kg daily. Data are expressed as mean ± SEM. Statistical significance was evaluated by applying ANOVA for independent variables: *** *p* < 0.001 vs. HC group, +++ *p* < 0.001 vs. AA group, + *p* < 0.05 vs. AA group.

The animals’ weights were initially recorded at the onset of the pilot experiment and subsequently every seven days thereafter. The variance was calculated between the weight measured on a specific day (7, 14, 21, and 28) and the weight on the first day. A notable decline in body weight within the untreated AA group was first observed on day 14 compared to the control group (AA *** *p* < 0.001 vs. HC group). This significant weight disparity persisted between the HC and AA groups through the experiment’s conclusion on day 28. A significant therapeutic impact was observed on all experimental days in the AA group treated with MTX at an oral dose of 0.3 mg/kg twice weekly (on days 14 and 21: MTX +++ *p* < 0.001 vs. AA group on day 28: MTX + *p* < 0.05). Astaxanthin, administered in a monotherapeutic regimen in both doses (AS1 and AS2), did not show any effect on the change in animal weight throughout the experiment (Figure 1).

##### The Change in the Hind Paw Volume of Experimental Animals

**Figure 2:**
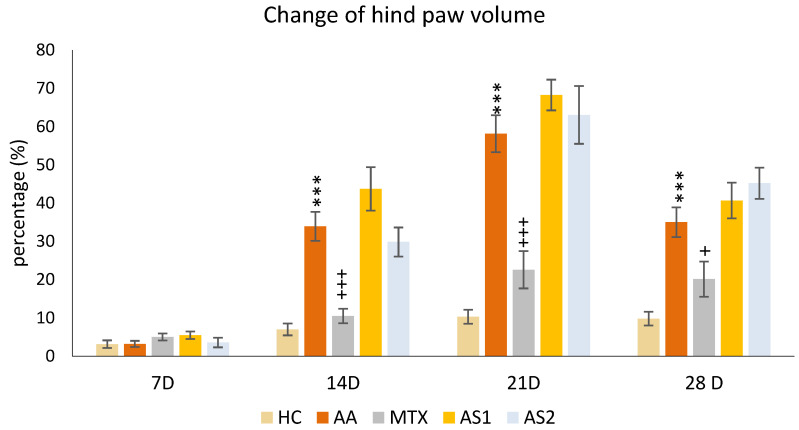
The presented data show the change in the hind paw volume measured on days 7, 14, 21, and 28 of the pilot experiment. The experimental animals were divided as follows: HC—healthy control group; AA—group of untreated rats with adjuvant arthritis (AA); MTX—rats with AA treated with methotrexate at a dose of 0.3 mg/kg twice weekly (MTX); AS1—group of rats with AA receiving astaxanthin of natural origin in the oral dose of 1 mg/kg daily; AS2—group of rats with AA receiving astaxanthin of natural origin in the oral dose of 5 mg/kg daily. Data are expressed as mean ± SEM. Statistical significance was evaluated by applying ANOVA for independent variables: *** *p* < 0.001 vs. HC group, +++ *p* < 0.001 vs. AA group, + *p* < 0.05 vs. AA group.

The animals’ percentual change in hind paw volume was measured on the given days of the pilot experiment: 7, 14, 21, and 28. A significant increase in the percentual change of hind paw volume in the untreated AA group was detected on days 14, 21, and 28 of the preliminary experiment (AA *** *p* < 0.001 vs. HC group). The monotherapy of MTX at a dose of 0.3 mg/kg twice weekly showed a significant decrease in this parameter on all experimental days (days 14 and 21: MTX +++ *p* < 0.001 vs. AA group on day 28: MTX + *p* < 0.05 vs. AA). On days 14 and 21, a worsening effect of astaxanthins on the volume of the hind limb was observed, with the smaller dose (AS1) exhibiting a more pronounced effect on worsening arthritis in the monitored parameter. However, this observation was not statistically proven (Figure 2).

#### 2.1.2. The Activity of GGT in the Spleen and Plasmatic Levels of IL-17A and MMP-9 on Day 28 in the Pilot Experiment

**Figure 3:**
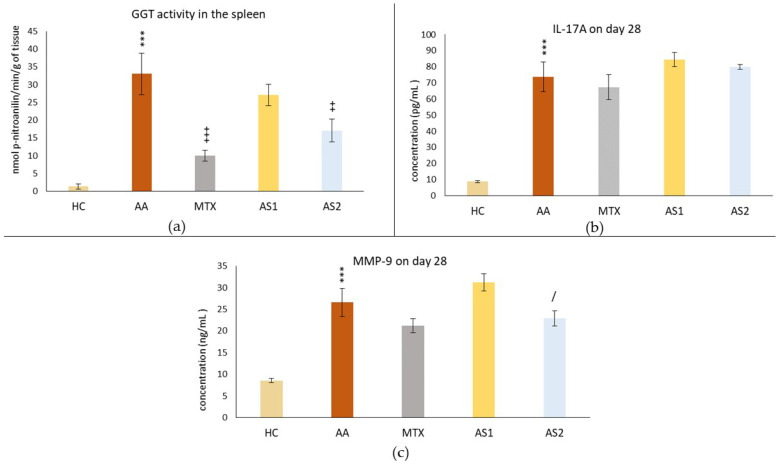
The activity of gamma-glutamyl transferase in the spleen (**a**), the level of IL-17A in the plasma (**b**), and the level of MMP-9 in the plasma (**c**) of the experimental animals measured on day 28 of the pilot experiment. The experimental animals were divided as follows: HC—healthy control group; AA—group of untreated rats with adjuvant arthritis (AA); MTX—rats with AA treated with methotrexate at a dose of 0.3 mg/kg twice weekly (MTX); AS1—group of rats with AA receiving astaxanthin of natural origin in the oral dose of 1 mg/kg daily; AS2—group of rats with AA receiving astaxanthin of natural origin in the oral dose of 5 mg/kg daily. Data are expressed as mean ± SEM. Statistical significance was evaluated by applying ANOVA for independent variables: *** *p* < 0.001 vs. HC group, +++ *p* < 0.001 vs. AA group, ++ *p* < 0.01 vs. AA group, / *p* < 0.05 vs. AS1 group.

Figure 3a shows the activity of gamma-glutamyl transferase (GGT) in the spleen, measured on day 28. A statistically significant difference was observed in the untreated AA group compared to the control group (AA *** *p* < 0.001 vs. HC group). The activity of GGT in the spleen in the group treated with MTX at a dose of 0.3 mg/kg twice weekly showed a significant decrease on day 28 compared to the untreated AA group (MTX +++ *p* < 0.001 vs. AA group) in this parameter. The AA group of animals receiving the lower dose of astaxanthin (1 mg/kg) (AS1) showed no statistically significant effect on this parameter, although a slight decrease in GGT activity was observed (AS1 *p* > 0.05 vs. AA). A group of animals with AA receiving astaxanthin of natural origin at a higher dose (5 mg/kg) (AS2) per day showed a statistically significant effect on the suppression of GGT activity compared to the untreated group (AS2 ++ *p* < 0.01 vs. AA).

Figure 3b shows the plasmatic level of IL-17A on day 28. Measuring the immunological parameter IL-17A, a statistically significant difference was observed in the untreated AA group compared to the control group (AA *** *p* < 0.001 vs. HC group). None of the monotherapies (MTX, AS1, and AS2) affected the IL-17A level in plasma compared to the untreated AA group on day 28 (MTX, AS1, and AS2 *p* > 0.05 vs. AA group). 

Figure 3c shows the plasmatic level of MMP-9 on day 28. The same pattern was seen with the IL-17A parameter. Administration of MTX on all experimental days was without a statistically confirmed therapeutic effect, although on all monitored days, a slight adjustment of MMP-9 level towards control values was observed (MTX *p* > 0.05 vs. AA). AS2 showed a better effect in lowering the MMP-9 level than AS1 (AS2 / *p* < 0.05). 

#### 2.1.3. The Plasmatic Level of Total Coenzyme Q_9_ (CoQ_9_) and the Level of Reduced CoQ_9_ in the Liver on Day 28

**Figure 4:**
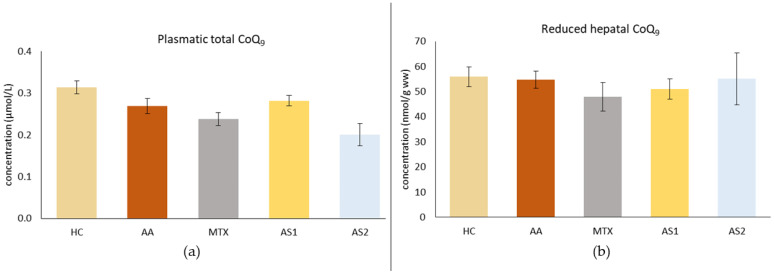
The presented data show the plasmatic level of total CoQ_9_ (**a**) and the level of reduced CoQ_9_ in the liver (**b**) of experimental animals measured on day 28. The experimental animals were divided as follows: HC—healthy control group; AA—group of untreated rats with adjuvant arthritis (AA); MTX—rats with AA treated with methotrexate at a dose of 0.3 mg/kg twice weekly (MTX); AS1—group of rats with AA receiving astaxanthin of natural origin in the oral dose of 1 mg/kg daily; AS2—group of rats with AA receiving astaxanthin of natural origin in the oral dose of 5 mg/kg daily. Data are expressed as mean ± SEM. Statistical significance was evaluated by applying ANOVA to independent variables.

Figure 4a shows the plasmatic level of total CoQ_9_ on day 28. As a result of the disease, a decrease in CoQ_9_ concentration was observed in the untreated AA group. Monotherapies showed no effect on reducing the plasma concentration of CoQ_9_ (AA, MTX, AS1, AS2 *p* > 0.05). Figure 4b shows the level of reduced CoQ_9_ in the liver on day 28. The disease did not affect this parameter. Administration of MTX tended to decrease the concentration of reduced CoQ_9_ in the liver, as did administration of AS1. AS2 tended to increase the concentration of this parameter. However, these observations were not statistically confirmed (MTX, AS1, and AS2 *p* > 0.05 vs. AA).

#### 2.1.4. The Hepatic Relative mRNA Expressions of Heme Oxygenase Subtype 1 (HO-1), Interleukin 1β (IL-1β), and Platelet-Activating Factor-Acetylhydrolase (PAF-AH) on Day 28

**Figure 5:**
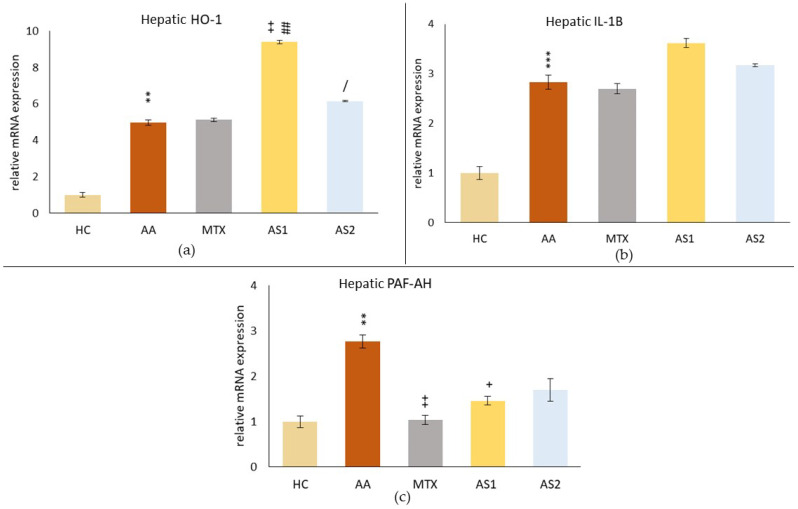
The evaluation of hepatic relative mRNA expressions of heme oxygenase subtype 1 (HO-1) (**a**), interleukin 1β (IL-1β) (**b**), and platelet-activating factor-acetylhydrolase (PAF-AH) (**c**) on day 28. The experimental animals were divided as follows: HC—healthy control group; AA—group of untreated rats with adjuvant arthritis (AA); MTX—rats with AA treated with methotrexate at a dose of 0.3 mg/kg twice weekly (MTX); AS1—group of rats with AA receiving astaxanthin of natural origin in the oral dose of 1 mg/kg daily; AS2—group of rats with AA receiving astaxanthin of natural origin in the oral dose of 5 mg/kg daily. Data are expressed as mean ± SEM. Statistical significance was evaluated by applying ANOVA for independent variables: ** *p* < 0.01 vs. HC group, *** *p* < 0.001 vs. HC group, + *p* < 0.05 vs. AA group, ++ *p* < 0.01 vs. AA group, ## *p* < 0.01 vs. MTX group, / *p* < 0.05 vs. AS1 group.

As a result of the disease, an increase in the hepatic relative mRNA expression of HO-1 in the AA group (AA ** *p* < 0.05 vs. HC) was observed (Figure 5a). Administration of MTX did not affect these levels (MTX *p* > 0.05 vs. AA). In contrast, administration of naturally occurring astaxanthin at a lower oral dose of 1 mg/kg (AS1) significantly increased the relative mRNA expression of HO-1 in the liver compared to the untreated AA animals (AS1 ++ *p* < 0.01 vs. AA) and compared to MTX (AS1 ## *p* < 0.01 vs. MTX). The higher dose of astaxanthin increased this parameter less than the lower dose (AS2 / *p* < 0.05 vs. AS1).

Figure 5b shows the relative mRNA expressions of IL-1β in the liver on day 28, showing a similar pattern as HO-1; only the monotherapies did not affect the relative mRNA expression of IL-1β in the liver (MTX, AS1, and AS2 *p* > 0.05). 

The relative mRNA expression of PAF-AH in the liver (Figure 5c) was significantly increased due to AA on day 28 (AA ** *p* < 0.01 vs. HC). All monotherapies affected the PAF-AH relative mRNA expression. However, only MTX (MTX ++ *p* < 0.01 vs. AA) and lower dose of astaxanthin (AS1) (AS1 + *p* < 0.05 vs. AA) had a statistically confirmed effect.

#### 2.1.5. The Activity of Antioxidant Enzymes (SOD, GPx, and CAT) in Erythrocytes and the Trolox Equivalent Antioxidant Capacity (TEAC) and Lipoperoxide (LPx) Levels in Plasma

**Figure 6:**
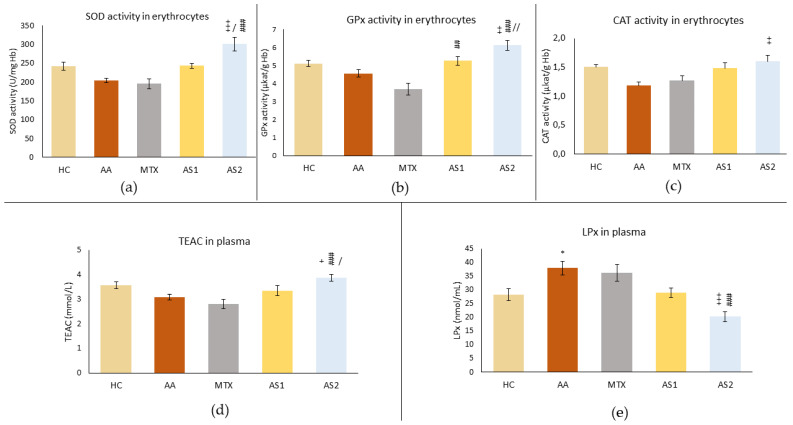
The effect of two doses of astaxanthin in monotherapy on parameters related to antioxidant status measured in erythrocytes—the superoxide dismutase (SOD), glutathione peroxidase (GPx), and catalase (CAT) activity (**a**–**c**), and in plasma—the Trolox equivalent antioxidant capacity (TEAC) and lipoperoxides (LPx) (**d**,**e**) of experimental animals on day 28. The experimental animals were divided as follows: HC—healthy control group; AA—group of untreated rats with adjuvant arthritis (AA); MTX—rats with AA treated with methotrexate at a dose of 0.3 mg/kg twice weekly (MTX); AS1—group of rats with AA receiving astaxanthin of natural origin in the oral dose of 1 mg/kg daily; AS2—group of rats with AA receiving astaxanthin of natural origin in the oral dose of 5 mg/kg daily. Data are expressed as mean ± SEM. Statistical significance was evaluated by applying ANOVA to independent variables: * *p* < 0.05 vs. HC group, + *p* < 0.05 vs. AA group, ++ *p* < 0.01 vs. AA group, +++ *p* < 0.001 vs. AA group, ## *p* < 0.01 vs. MTX group, ### *p* < 0.001 vs. MTX group, / *p* < 0.05 vs. AS1 group, // *p* < 0.01 vs. AS1 group.

Figure 6a shows the superoxide dismutase (SOD) activity in erythrocytes on day 28. As a consequence of AA, a reduced activity of this enzyme was observed in untreated rats. MTX also reduced the activity of SOD. Conversely, AS1 tended to increase activity to the level seen in healthy controls. However, these observations were not confirmed statistically (AA *p* > 0.05 vs. HC, MTX and AS1 *p* > 0.5 vs. AA). A higher dose of astaxanthin significantly increased SOD activity on day 28 compared to the untreated group (AS2 +++ *p* < 0.001 vs. AA) and other monotherapies (AS2 / *p* < 0.05 vs. AS1 and AS2 ### *p* < 0.001 vs. MTX).

Similar results to SOD were achieved in CAT activity in erythrocytes (Figure 6b). AS2 had a more significant effect compared to AS1 (AS2 ++ *p* < 0.01 vs. AS1).

A slight decrease in GPx activity was observed due to the disease in the AA group (Figure 6c). MTX tended to decrease GPx activity, but these observations were not proved statistically. Conversely, both astaxanthins (AS1 and AS2) increased GPx activity compared to MTX (AS1 ## *p* < 0.01 vs. MTX and AS2 ### *p* < 0.01 vs. MTX). The effect of a higher dose of astaxanthin significantly increased GPx activity during AA (AS2 ++ *p* < 0.01 vs. AA), and this positive effect was higher compared to the effect of AS1 (AS2 // *p* < 0.01 vs. AS1). 

The antioxidant capacity equivalent to Trolox (TEAC) was determined on day 28 (Figure 6d). The decrease in antioxidant capacity was observed in the AA group and due to the administration of MTX, while AS1 tended to increase this capacity. However, these observations did not prove to be statistically significant. Administration of AS2 caused a statistically significant increase in antioxidant capacity compared to AA (AS2 + *p* < 0.05), MTX (AS2 ### *p* < 0.001), and AS1 (AS2 / *p* < 0.05).

The LPx level was measured on day 28 in plasma (Figure 6e). As a result of AA, increased activity of this parameter was observed (AA * *p* < 0.05 vs. HC), while MTX also increased it. Conversely, AS1 tended to reduce LPx to physiological levels. However, these observations were not confirmed statistically (MTX and AS1 *p* > 0.5 vs. AA). The higher dose of astaxanthin significantly reduced the level of LPx on day 28 compared to the untreated group and to the MTX group (AS2 +++ *p* < 0.001 vs. AA and ### *p* < 0.001 vs. MTX).

Based on the results of the first experiment with two doses of astaxanthin (1 and 5 mg/kg daily), we expected a more significant therapeutic action. Thus, according to the literature findings, we selected a dose as high as 20 mg/kg daily for the pivotal experiment. The doses of β-cryptoxanthin and β-carotene were also selected according to the literature.

### 2.2. Results from the Pivotal Experiment

#### 2.2.1. Biometric Results from the Pivotal Experiment 

##### The Change in the Weight of the Animals during the Pivotal Experiment

**Figure 7:**
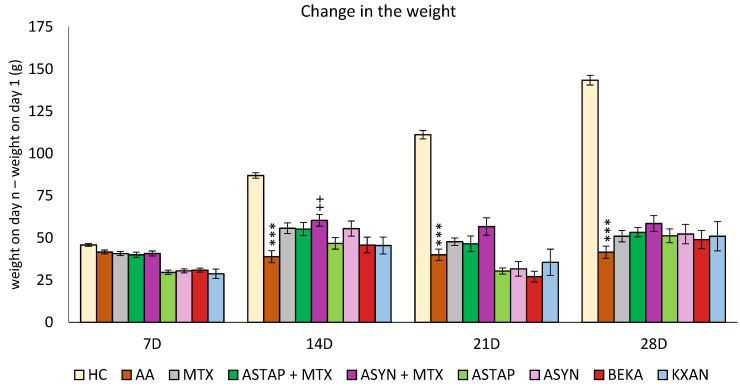
The presented data show the change in animal body weight measured on days 7, 14, 21, and 28 of the pivotal experiment. The experimental animals were divided as follows: HC—healthy control group; AA—group of untreated controls with adjuvant arthritis (AA); MTX—rats with AA treated with methotrexate (MTX) at a dose of 0.3 mg/kg twice weekly; ASTAP—group of rats with AA receiving astaxanthin of natural origin in the oral dose of 20 mg/kg daily, ASTAP + MTX—group of rats with AA receiving astaxanthin of natural origin in the oral dose of 20 mg/kg daily and in a combination with MTX at a dose of 0.3 mg/kg twice weekly; ASYN—group of rats with AA receiving synthetic astaxanthin in the oral dose of 20 mg/kg daily; ASYN + MTX—group of rats with AA receiving synthetic astaxanthin in the oral dose of 20 mg/kg daily and in combination with MTX at a dose of 0.3 mg/kg twice weekly; BEKA—group of rats with AA receiving β-carotene in the oral dose of 20 mg/kg daily; KXAN—group of rats with AA receiving β-cryptoxanthin in the oral dose of 10 μg/kg daily. Data are expressed as mean ± SEM. Statistical significance was evaluated using ANOVA for independent variables: *** *p* < 0.001 vs. HC group, ++ *p* < 0.01 vs. AA group.

The animals’ weight was measured at the beginning of the pivotal experiment and then every seven days. The difference between the weight measured on a given day (7, 14, 21 and 28) minus the weight on the first day was assessed. A significant decrease in body weight in the untreated AA group was first detected on day 14 (AA *** *p* < 0.001 vs. HC group) and lasted until the end of the experiment on day 28. Only ASYN + MTX combined therapy (++ *p* < 0.01 vs. AA) showed significant weight gain on day 14, and this weight gain trend persisted until the end of the experiment. No other statistically significant therapeutic effect was demonstrated in this biometric parameter for any monotherapy group (MTX, ASTAP, ASYN, BEKA, KXAN) or combination therapy group with MTX (Figure 7).

However, on day 28, all therapies had a positive effect in correcting the weight loss caused by AA. 

##### The Change in Hind Paw Volume of the Animals during the Pivotal Experiment

**Figure 8:**
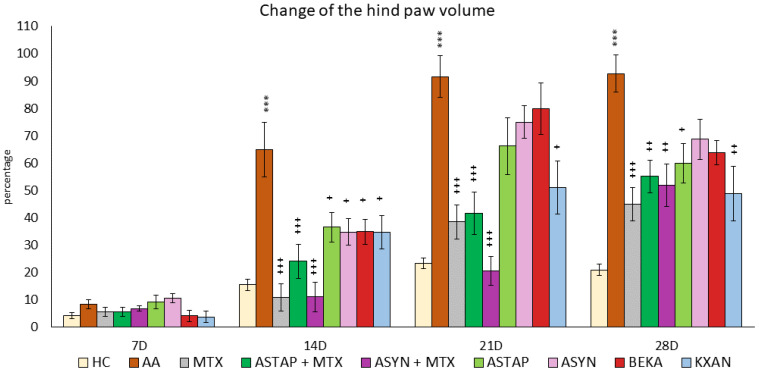
The presented data show the percentual change of the hind paw volume of experimental animals measured on 14, 21, and 28 days during the pivotal experiment. HC—healthy control group; AA—group of untreated controls with adjuvant arthritis (AA); MTX—rats with AA treated with methotrexate (MTX) at a dose of 0.3 mg/kg twice weekly; ASTAP—group of rats with AA receiving astaxanthin of natural origin in the oral dose of 20 mg/kg daily; ASTAP + MTX—group of rats with AA receiving astaxanthin of natural origin in the oral dose of 20 mg/kg daily and in a combination with MTX at a dose of 0.3 mg/kg twice weekly; ASYN—group of rats with AA receiving synthetic astaxanthin in the oral dose of 20 mg/kg daily; ASYN + MTX—group of rats with AA receiving synthetic astaxanthin in the oral dose of 20 mg/kg daily and in combination with MTX at a dose of 0.3 mg/kg twice weekly; BEKA—group of rats with AA receiving β-carotene in the oral dose of 20 mg/kg daily; KXAN—group of rats with AA receiving β-cryptoxanthin in the oral dose of 10 μg/kg daily. Data are expressed as mean ± SEM. Statistical significance was evaluated using ANOVA for independent variables: *** *p* < 0.001 vs. HC group, + *p* < 0.05 vs. AA group, ++ *p* < 0.01 vs. AA group, +++ *p* < 0.001 vs. AA group.

On day 7, no differences were observed between the experimental groups. However, during the remaining experimental days (14, 21, and 28), the untreated AA group showed a significant increase in the percentage change in hind paw volume compared to the HC group, indicating the presence of inflammation (AA *** *p* < 0.001 vs. HC). MTX monotherapy caused a significant reduction in the volume of the hind paw during days 14, 21, and 28 (MTX +++ *p* < 0.001 vs. AA). Among the monotherapies (ASTAP, ASYN, BEKA, and KXAN), all administered substances showed statistically significant effects on reducing the volume of the hind paw on day 14 (ASTAP, ASYN, BEKA, KXAN + *p* < 0.05 vs. AA), on day 21 only β-cryptoxanthin (KXAN + *p* < 0.05 vs. AA), and on day 28 ASTAP (+ *p* < 0.05 vs. AA) and KXAN (++ *p* < 0.01 vs. AA) were effective. Combined therapies with MTX were effective on experimental days 14, 21, and 28, with the ASYN + MTX combination showing the most significant reduction in swelling. On day 14, ASYN + MTX had the same efficacy as MTX, but on day 21, the ASYN + MTX combination therapy was the most evident in reducing this parameter (Figure 8).

##### Arthritic Score of the Animals during the Pivotal Experiment

**Figure 9:**
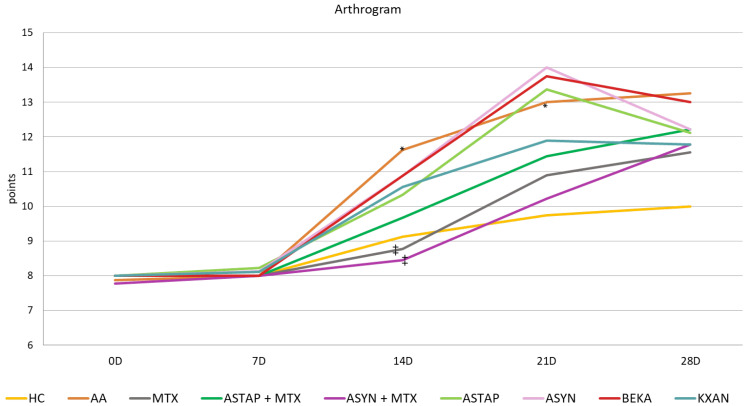
The presented data show the arthrogram by using a line graph. The experimental animals were divided as follows: HC—healthy control group; AA—group of untreated controls with adjuvant arthritis (AA); MTX—rats with AA treated with methotrexate (MTX) at a dose of 0.3 mg/kg twice weekly; ASTAP—group of rats with AA receiving astaxanthin of natural origin in the oral dose of 20 mg/kg daily; ASTAP + MTX—group of rats with AA receiving astaxanthin of natural origin in the oral dose of 20 mg/kg daily and in a combination with MTX at a dose of 0.3 mg/kg twice weekly; ASYN—group of rats with AA receiving synthetic astaxanthin in the oral dose of 20 mg/kg daily; ASYN + MTX—group of rats with AA receiving synthetic astaxanthin in the oral dose of 20 mg/kg daily and in combination with MTX at a dose of 0.3 mg/kg twice weekly; BEKA—group of rats with AA receiving β-carotene in the oral dose of 20 mg/kg daily; KXAN—group of rats with AA receiving β-cryptoxanthin in the oral dose of 10 μg/kg daily. Data are expressed as mean ± SEM. Statistical significance was evaluated using ANOVA for independent variables: * *p* < 0.05 vs. HC group, ++ *p* < 0.01 vs. AA group.

The arthrogram was evaluated based on the points obtained during the experiment. The effect of arthritis was confirmed by the worsening of the arthritis score on all experimental days, statistically on days 14 and 21 (AA * *p* < 0.05 vs. HC). MTX modified the arthrogram values throughout the experiment, statistically significantly on day 14 (MTX ++ *p* < 0.01 vs. AA). Both combined therapies were similar in effect, as was KXAN in monotherapy. Efficacy decreased in the order ASYN + MTX, MTX, ASTAP + MTX, and KXAN. The most effective combination was ASYN + MTX on day 14 (++ *p* < 0.01 vs. AA) and KXAN in monotherapy on day 28. The monotherapeutic treatment, except for KXAN, showed a slightly worsening effect on the monitored parameter, especially on the 21st day. However, on days 14 and 28, a trend towards improvement was noted. Overall, BEKA was the least effective (Figure 9).

#### 2.2.2. Activity of Gamma-Glutamyl Transferase (GGT) in the Spleen on Day 28 

**Figure 10:**
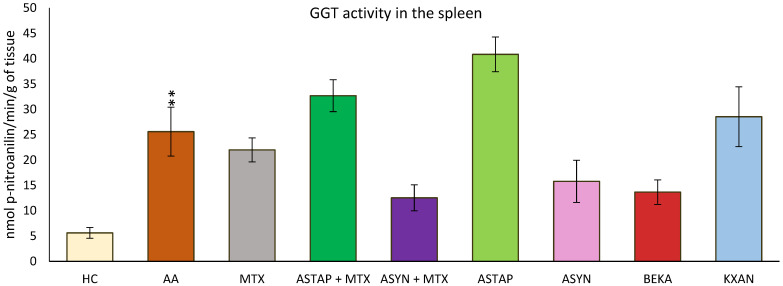
The presented data show the activity of GGT in the spleen of experimental animals measured on day 28 of the pivotal experiment. The experimental animals were divided as follows: HC—healthy control group; AA—group of untreated controls with adjuvant arthritis (AA); MTX—rats with AA treated with methotrexate (MTX) at a dose of 0.3 mg/kg twice weekly; ASTAP—group of rats with AA receiving astaxanthin of natural origin in the oral dose of 20 mg/kg daily; ASTAP + MTX—group of rats with AA receiving astaxanthin of natural origin in the oral dose of 20 mg/kg daily and in a combination with MTX at a dose of 0.3 mg/kg twice weekly; ASYN—group of rats with AA receiving synthetic astaxanthin in the oral dose of 20 mg/kg daily; ASYN + MTX—group of rats with AA receiving synthetic astaxanthin in the oral dose of 20 mg/kg daily and in combination with MTX at a dose of 0.3 mg/kg twice weekly; BEKA—group of rats with AA receiving β-carotene in the oral dose of 20 mg/kg daily; KXAN—group of rats with AA receiving β-cryptoxanthin in the oral dose of 10 μg/kg daily. Data are expressed as mean ± SEM. Statistical significance was evaluated using ANOVA for independent variables: ** *p* < 0.01 vs. HC group.

GGT activity in the spleen was measured at the end of the experiment (day 28). As a result of the disease, increased GGT activity was observed in the untreated AA group (AA ** *p* < 0.01 vs. HC). The ASYN and BEKA monotherapies tended to decrease the activity of this parameter, while among the combined therapies, ASYN + MTX was more effective than ASTAP + MTX. The remaining monotherapeutic groups (MTX and KXAN) did not modify the increased GGT activity; moreover, the ASTAP + MTX and ASTAP groups tended to increase GGT activity in the spleen compared to the untreated AA group, although these observations were not statistically confirmed (Figure 10). 

#### 2.2.3. Plasmatic Levels of IL-17A on Days 14 and 28

**Figure 11:**
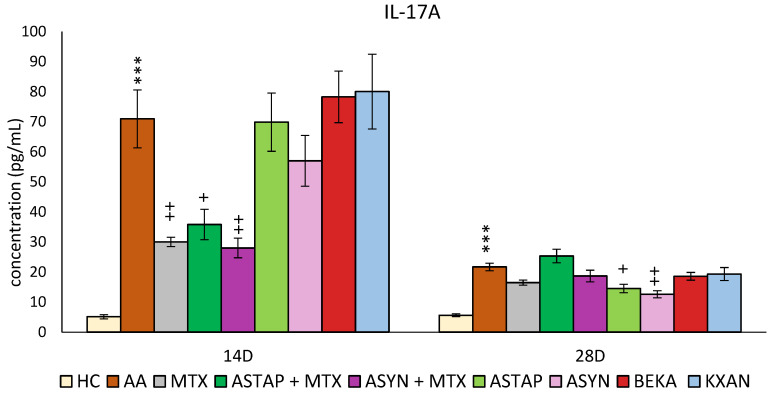
The presented data show the levels of IL-17A in the plasma of experimental animals measured on days 14 and 28 of the pivotal experiment. The experimental animals were divided as follows: HC—healthy control group; AA—group of untreated controls with adjuvant arthritis (AA); MTX—rats with AA treated with methotrexate (MTX) at a dose of 0.3 mg/kg twice weekly; ASTAP—group of rats with AA receiving astaxanthin of natural origin in the oral dose of 20 mg/kg daily; ASTAP + MTX—group of rats with AA receiving astaxanthin of natural origin in the oral dose of 20 mg/kg daily and in a combination with MTX at a dose of 0.3 mg/kg twice weekly; ASYN—group of rats with AA receiving synthetic astaxanthin in the oral dose of 20 mg/kg daily; ASYN + MTX—group of rats with AA receiving synthetic astaxanthin in the oral dose of 20 mg/kg daily and in combination with MTX at a dose of 0.3 mg/kg twice weekly; BEKA—group of rats with AA receiving β-carotene in the oral dose of 20 mg/kg daily; KXAN—group of rats with AA receiving β-cryptoxanthin in the oral dose of 10 μg/kg daily. Data are expressed as mean ± SEM. Statistical significance was evaluated using ANOVA for independent variables: *** *p* < 0.001 vs. HC group, + *p* < 0.05 vs. AA group, ++ *p* < 0.01 vs. AA group.

On days 14 and 28, the concentration of IL-17A in the plasma was determined (Figure 11). On day 14, a significant increase in IL-17A concentration was observed due to AA (AA *** *p* < 0.001 vs. HC). Administration of MTX significantly reduced these levels (MTX ++ *p* < 0.01 vs. AA). Other monotherapies (ASTAP, ASYN, BEKA, and KXAN) did not work in favor of reducing the levels of this parameter on day 14. Combination therapies reduced IL-17A concentrations on day 14, more significantly than ASYN + MTX than ASTAP + MTX (ASYN + MTX ++ *p* < 0.01 vs. AA, ASTAP + MTX + *p* < 0.05 vs. AA). 

On day 28, IL-17A levels remained elevated due to AA (AA*** *p* < 0.001 vs. HC). Surprisingly, on this day, only ASYN and ASTAP monotherapies were shown to reduce IL-17A levels (ASYN ++ *p* < 0.01 vs. AA, ASTAP + *p* < 0.05 vs. AA). MTX, BEKA, and KXAN monotherapies did not affect IL-17A levels on day 28, similarly to the combined ASYN + MTX and ASTAP + MTX therapies (Figure 11).

#### 2.2.4. Plasmatic Levels of MMP-9 on Days 14 and 28 

**Figure 12:**
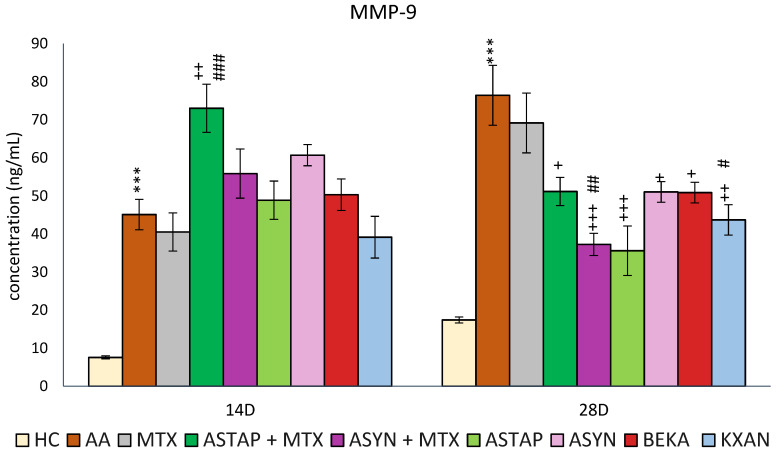
The presented data show the concentration of MMP-9 in the plasma of experimental animals measured on days 14 and 28 of the pivotal experiment. The experimental animals were divided as follows: HC—healthy control group; AA—group of untreated controls with adjuvant arthritis (AA); MTX—rats with AA treated with methotrexate (MTX) at a dose of 0.3 mg/kg twice weekly; ASTAP—group of rats with AA receiving astaxanthin of natural origin in the oral dose of 20 mg/kg daily; ASTAP + MTX—group of rats with AA receiving astaxanthin of natural origin in the oral dose of 20 mg/kg daily and in combination with MTX at a dose of 0.3 mg/kg twice weekly; ASYN—group of rats with AA receiving synthetic astaxanthin in the oral dose of 20 mg/kg daily; ASYN + MTX—group of rats with AA receiving synthetic astaxanthin in the oral dose of 20 mg/kg daily and in combination with MTX at a dose of 0.3 mg/kg twice weekly; BEKA—group of rats with AA receiving β-carotene in the oral dose of 20 mg/kg daily; KXAN—group of rats with AA receiving β-cryptoxanthin in the oral dose of 10 μg/kg daily. Data are expressed as mean ± SEM. Statistical significance was evaluated using ANOVA for independent variables: *** *p* < 0.001 vs. HC group, + *p* < 0.05 vs. AA group, ++ *p* < 0.01 vs. AA group, +++ *p* < 0.001 vs. AA group, # *p* < 0.05 vs. MTX, ## *p* < 0.01 vs. MTX, ### *p* < 0.001 vs. MTX.

Plasma MMP-9 levels were evaluated on days 14 and 28. MMP-9 levels were increased as a consequence of the disease similarly on days 14 and 28 (AA *** *p* < 0.001 vs. HC). However, on day 28, the measured MMP-9 concentration was higher compared to day 14. On both experimental days, MTX had a minimal therapeutic effect on this parameter. KXAN had a similar effect to MTX on day 14, while all other monotherapies and combined therapies had no effect. Notably, ASTAP + MTX levels were increased compared to the untreated AA group and MTX monotherapy. On day 28, all monotherapies (apart from MTX) and combined therapies reduced MMP-9 levels, with the most effective combination being ASYN + MTX (+++ *p* < 0.001 vs. AA, ## *p* < 0.01 vs. MTX), followed by KXAN (++ *p* <0.01 vs. AA, # *p* < 0.05 vs. MTX) and ASTAP (+++ *p* < 0.001 vs. AA). The other three, ASTAP + MTX, ASYN, and BEKA, had similar effects (+ *p* < 0.05) (Figure 12).

#### 2.2.5. Levels of Reduced CoQ_9_ in the Liver

**Figure 13:**
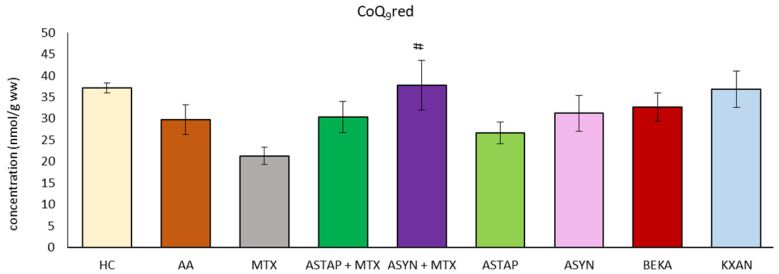
The presented data show the level of the reduced coenzyme Q_9_ (CoQ_9_red) in the liver of experimental animals measured on day 28 of the pivotal experiment. The experimental animals were divided as follows: HC—healthy control group; AA—group of untreated controls with adjuvant arthritis (AA); MTX—rats with AA treated with methotrexate (MTX) at a dose of 0.3 mg/kg twice weekly; ASTAP—group of rats with AA receiving astaxanthin of natural origin in the oral dose of 20 mg/kg daily; ASTAP + MTX—group of rats with AA receiving astaxanthin of natural origin in the oral dose of 20 mg/kg daily and in a combination with MTX at a dose of 0.3 mg/kg twice weekly; ASYN—group of rats with AA receiving synthetic astaxanthin in the oral dose of 20 mg/kg daily; ASYN + MTX—group of rats with AA receiving synthetic astaxanthin in the oral dose of 20 mg/kg daily and in combination with MTX at a dose of 0.3 mg/kg twice weekly; BEKA—group of rats with AA receiving β-carotene in the oral dose of 20 mg/kg daily; KXAN—group of rats with AA receiving β-cryptoxanthin in the oral dose of 10 μg/kg daily. Data are expressed as mean ± SEM. Statistical significance was evaluated using ANOVA for independent variables: # *p* < 0.05 vs. MTX.

The concentration of reduced coenzyme Q_9_ (CoQ_9_red) was determined in the liver on day 28 of the experiment (Figure 13). Due to the AA, we observed reduced levels of CoQ_9_red. During MTX administration, the concentration of CoQ_9_red in the liver decreased. All other applications reached higher levels than MTX, and the ASYN + M combination was statistically significant (# *p* < 0.05 vs. MTX). Correction of the decreased level occurring under the influence of AA and return towards healthy control values were observed only after administration of KXAN (*p* >0.05) and ASYN + M.

## 3. Discussion

Adjuvant arthritis (AA) is a well-established model for studying rheumatoid arthritis (RA) due to its similarities in pathogenesis [38] and immune responses [39]. Astaxanthin, a potent carotenoid with antioxidant [40] and anti-inflammatory properties [41], has gained a lot of attention for its therapeutic effects in various inflammatory conditions [42,43,44]. In this comprehensive scientific discussion, the authors analyze the results of two experiments (a pilot and a pivotal) investigating the in vivo effects of astaxanthin on the course of experimental arthritis, along with its combination with methotrexate (MTX), the standard drug for RA patients [45,46]. The focus of the pilot experiment was to compare the different doses of astaxanthin in monotherapy compared to MTX treatment. In the following pivotal experiment, authors compared different sources of astaxanthin in monotherapy and in combination with MTX, along with other potent carotenoids such as β-carotene (BEKA) and β-cryptoxanthin (KXAN) in monotherapy.

In the pilot experiment, the organic astaxanthin isolated from *Blakeslea trispora* was used in monotherapy in doses of 1 mg/kg and 5 mg/kg of b.w., respectively. The higher dose of astaxanthin (AS2) tended to influence multiple parameters in a positive aspect (change of the body weight on day 14 (Figure 1) and amelioration of the hind paw volume on day 14 (Figure 2). AS2 also showed a significant effect on lowering the activity of GGT in the spleen (Figure 3a) and tended to lower the MMP-9 concentration on day 28 (Figure 3c). Additionally, AS2 lowered the relative mRNA expression of HO-1 in the liver (Figure 5a) compared to AS1. On the contrary, AS2 increased the activity of SOD, GPx, and CAT in erythrocytes (Figure 6a–c) and TEAC (Figure 6d) concentration in plasma. Moreover, AS2 caused a decline in plasmatic LPx levels (Figure 6e). 

Looking at the pilot experimental biometric results, untreated AA rats exhibited a significant decline in body weight compared to healthy controls, indicative of disease progression, as shown in Figure 1, which is in line with previous findings [35,47]. Similarly, this result has already been described in various other studies with the same or related AA rat model [48,49,50]. While MTX treatment showed obvious therapeutic benefits, astaxanthin monotherapy did not significantly impact the weight change (Figure 1). This might indicate that astaxanthin has negligible or no effect on body fat and muscle metabolism. This result was observed again in the second experiment (Figure 7) as well. Moreover, it was shown that prolonged astaxanthin supplementation does not increase fat oxidative capacity in humans [51]. However, other authors have described that astaxanthin improves muscle lipid metabolism [52]. Astaxanthin supplementation in the diet has positive effects for the modification of the symptoms associated with obesity in animals: a significant decrease in the weight of adipose tissue was found, systolic pressure was reduced, insulin sensitivity was improved, glycemia was reduced, as well as the levels of cholesterol, triacylglycerols (TAG), alanine aminotransferase (ALT), and aspartate aminotransferase (AST) in the liver [53]. Discrepancies in these findings may be attributed to differences in models, parameters investigated, assessment methods, co-treatments, and environmental conditions. As suggested by Radice et al. (2021) in their meta-analysis, further investigation is warranted to draw definitive conclusions [53].

During this pilot experiment, another biometric parameter, hind paw volume, was assessed (Figure 2). Considering the result, AA rats displayed a significant increase in hind paw volume, indicative of inflammation, which was alleviated by MTX treatment. A similar pattern of untreated AA and MTX at an oral dose of 0.3 mg/kg twice a week was observed during the pivotal experiment (Figure 8). These findings are typical for the model of AA and were also confirmed in our previous experiments [54]. However, the treatment effect represented by astaxanthin monotherapy in two doses (1 mg/kg and 5 mg/kg) was not confirmed statistically (Figure 2). This might indicate that the doses of astaxanthin in the pilot experiment were not sufficient to achieve a significant antirheumatic effect. Therefore, we used astaxanthin from the same source but at a higher dose of 20 mg/kg of b.w. and also in combination therapy with MTX in the pivotal experiment. There were other studies evaluating astaxanthin in higher dosages (20–144 mg/kg) [55,56]. We have found that higher doses of natural and synthetic AS have a significant effect on reducing the hind paw volume in monotherapy and in combination with MTX.

While organic and synthetic astaxanthin share the same chemical formula, there are differences between them: esterification, stereochemistry, and the fact that organic astaxanthin can occur in a complex with other carotenoids [57]. Two asymmetric carbon atoms can be found in the astaxanthin molecule at positions 3 and 3′. As a result, three different optical isomers or enantiomers are possible: (3R, 3′R); (3S, 3′S); and (3R, 3′S). The most prevalent isomers in nature have chirality (3S, 3′S), or (3R, 3′R); among these, the former has the highest reported antioxidant activity [3,48]. Synthetic astaxanthin consists of (3S, 3′S), (3R, 3′S), (3S, 3′R), and (3R, 3′R), in a 1:2:2:1 ratio, respectively [4], and exists only in the non-esterified form [57]. Based on their different properties, astaxanthin from organic and synthetic origins may exert different effects [58]. Organic astaxanthin proved to be 14–65 times more potent at eliminating free radicals when compared to astaxanthin of synthetic origin in in vitro conditions [57]. However, our in vivo preclinical study does not support this finding. 

The astaxanthin isolated from *Blakeslea trispora* was evaluated in vitro using the DPPH assay and found to have a higher free radical quenching ability compared to alpha-tocopherol, butylhydroxytoluene, butylhydroxyanisole, gallic acid, and Trolox [21]. 

In our in vivo AA model, astaxanthin demonstrated significant antioxidant potential even at a lower dose (5 mg/kg), as evidenced by increased activity of antioxidant enzymes (SOD, GPx, and CAT) and improved markers of oxidative stress, including increased TEAC and decreased LPx compared to the untreated AA group (Figure 6).

Astaxanthin and its isolated esters showed hepatoprotective and antioxidant activity by restoring antioxidant enzyme levels (catalase (CAT), glutathione peroxidase (GPx), superoxide dismutase (SOD), and lipid peroxidase) in carbon tetrachloride-treated rats compared to controls [24]. During ethanol-induced gastric damage, oxygen radicals are released. Pretreatment with astaxanthin fractions (total carotenoid and astaxanthin esters) obtained from *H. pluvialis* at a concentration of 100, 250, or 500 μg/kg markedly boosted antioxidant enzymes CAT, SOD, and GPx in the homogenate of rat stomach tissue of Wistar rats with ethanol-induced stomach ulcers. In this study, astaxanthin was found to be able to inhibit the enzyme 15-lipooxygenase. Low-density lipoprotein (LDL) oxidation is believed to be mediated by 15-lipooxygenase, which could be important for inflammatory reactions in ulcerative disease damage [26]. In the C57BL/KsJ-*db*/*db* (*db*/*db*) obese mice model of azoxymethane (AOM)-induced colonic premalignant lesions, 8 weeks of 200 ppm astaxanthin administration in diet significantly decreased the levels of OS indicators, 8-hydroxyguanosine (8-OHdG), and the reactive oxygen metabolites (d-ROMs) in the urine and serum, while raising the expression of mRNA for the antioxidant enzymes GPx1, SOD1, and CAT in the colonic mucosa of AOM-treated db/db mice. Astaxanthin dramatically reduced the expression levels of mRNA for IL-1β, IL-6, F4/80, C-C Motif Chemokine Ligand 2 (CCL2), and chemokine (C-X-C motif) ligand 2 (CXCL2) in the colonic mucosa of mice treated with AOM. In the colonic epithelium, dietary treatment with astaxanthin also led to decreased NF-κB and proliferating cell nuclear antigen (PCNA)-positive cells, which were elevated by exposure to AOM [27]. Moreover, astaxanthin proved more effective than vitamin E in preventing and treating nonalcoholic steatohepatitis in mice fed 0.02% astaxanthin for 12 weeks [28].

The enzyme gamma-glutamyl transferase (GGT) is found on the cell surfaces of numerous body tissues and is believed to be one of the pathogenic factors causing inflammatory processes. In experimental arthritis models, such as the collagen-induced arthritis (CIA) model, increased GGT expression and activity in joint tissue is a valid experimental indication of synovial inflammation. Anti-GGT antibodies are proposed as novel therapeutic agents that neutralize GGT, resulting in lowering osteoclast production and joint degeneration in RA patients [59,60]. The GGT activity in the spleen was chosen based on our previous results [61]. MTX and a higher dose of astaxanthin (AS2) significantly decreased GGT activity in the spleen (Figure 3a), indicating a reduction in the inflammatory response. The significant role of MTX in reducing GGT activity in the spleen was also demonstrated in previous experiments [62]. Regarding the significant effect of AS2 (Figure 3a), indirect evidence was described by Ghlissi et al. (2014), who examined the efficacy of natural astaxanthin (20 mg/kg) in preventing colistin-induced nephrotoxicity in the rat model. The authors concluded that astaxanthin restored urine γ-glutamyl-transferase (GGT) levels [63]. We may hypothesize that this might have a similar mechanism in our experiment. However, the conclusion should be based on further investigation.

None of the astaxanthin treatments (AS1 and AS2) in lower doses significantly impacted increased IL-17A or MMP-9 levels (Figure 3b,c). IL-17A is produced by activated adaptive and innate immune cells [64] and contributes to the inflammatory changes seen during RA. Various studies showed the enhancing effect of IL-17 in inducing the production of proinflammatory cytokines IL-1β, TNF-α, IL-6, and IL-8 [65]. Enzymes known as MMPs are generated by activated macrophages and fibroblasts in reaction to proinflammatory cytokines, including TNF-α and IL-1. It has been demonstrated that they contribute to the articular tissue degradation in RA [66,67]. We hypothesize that astaxanthin failed to show its effect in the pilot experiment due to its low 1 and 5 mg/kg doses.

Coenzyme Q_9_ (CoQ_9_) is a dominant form of CoQ in rats. It occurs in reduced and oxidized forms [68]. Total CoQ_9_ (CoQ_9_Tot) in plasma includes both forms; it reflects the organism’s bioenergetic and antioxidant capacity. AA tended to decrease levels of CoQ_9_Tot in plasma on day 28 (Figure 4a); however, it was not confirmed statistically. Our previous findings describe the level of CoQ_9_ in plasma as being lower during AA [68,69]. There are very few scientific articles in the literature monitoring the plasma CoQ_10_ levels in RA patients. In one study, authors found that the level of CoQ_10_ in RA patients was without significant change compared to healthy subjects [70], similar to our current results. As the change in plasmatic CoQ_10_ levels in RA patients and in animals is still an open question, it is important to continue further research to find appropriate answers.

A reduced form of CoQ_9_ (CoQ_9_red) in liver tissue may protect against oxidative stress [71]. In a model of hepatotoxic damage in Wistar rats, a significant decrease in the concentration of CoQ_9_red in the liver of the animals was observed [69]. However, no changes in CoQ_9_red concentrations have been detected in arthritic or treated animals (Figure 4b). In the pivotal experiment (Figure 13), only combined therapy (ASYN + MTX) was beneficial compared to MTX in monotherapy.

The gene expression of the antioxidant enzyme HO-1 (Figure 5a) was increased in the AA group to a similar extent as in our previous experiment [72]. The expression was more elevated at lower AS1 concentrations. AS2 did not reduce the expression of HO-1, similar to MTX. Gene expression of HO-1 is controlled by three transcription factors: Nrf2 (a sensor for oxidative stress), NF-κB, and AP-1 (proinflammatory transcription factors), so it is difficult to determine the reason for the shown increase of HO-1 in the AA group. Gene expression of the proinflammatory cytokine, such as IL-1β (Figure 5b), which is under the control of NF-κB, was also increased by AS1. Other studies have described a regulative effect of astaxanthin on Nrf2 activation [73,74] as well. 

Similarly, as in our previous experiments, mRNA expression of IL-1β was elevated in the liver of arthritic animals [75]. Astaxanthin in both dosages slightly increased the expression of IL-1β (Figure 5b). Moreover, there was no apparent reduction in IL-1β mRNA expression in the liver following MTX administration. This aligns with previous studies on the role of MTX in numerous cell types, such as human peripheral blood mononuclear cells and mouse peritoneal and spleen cells [76]. However, MTX demonstrated a specific mechanism of inhibiting the activity of IL-1β, which prevents IL-1β from binding to the IL-1β receptor found in the membrane of peripheral blood cells, such as monocytes, lymphocytes, and granulocytes [77].

In experiments with acute phase response (APR) in rodents, lipopolysaccharide (LPS) significantly induced the mRNA expression of PAF-AH in the liver and spleen and increased the serum PAF-AH activity [78,79]. In RA patients, the total serum PAF-AH activity decreased in most studies [80,81] or even increased in some studies [55]. In rodents, most of the PAF-AH activity is HDL-bound, and in humans, 70% of the enzyme is bound to LDL and 30% to HDL. The decrease of PAF-AH activity under inflammatory conditions is connected to the decrease of LDL particles [80], whereas HDL-bound enzyme activity increases [79]. No data about the mRNA expression of this enzyme in the rat liver in the model of adjuvant arthritis or under chronic inflammatory conditions are known. In our experiment, we report for the first time that PAF-AH gene mRNA expression was significantly increased in the liver of AA rats, as was the expression of the proinflammatory cytokine IL-1β. Both concentrations of astaxanthin and MTX (AS1 and MTX significantly) decreased PAF-AH mRNA (Figure 5c), which does not correlate with the IL-1β mRNA levels (Figure 5b). IL-1β and TNFα, respectively, were shown to have only a modest impact on the mRNA levels in the APR model in rodents, indicating the role of some other proinflammatory mediator in the activation of transcription [79]. Since the sensitive marker of inflammation, PAF-AH [82], was reduced by astaxanthin administration, we hypothesize that astaxanthin has reduced inflammation in our experiment, but further studies are needed to elucidate the mechanism or the specific inhibited proinflammatory mediator. The reduction of PAF-AH by astaxanthin might reduce cardiovascular risk in RA patients, as PAF-AH levels were positively correlated with the severity of atherosclerosis in RA patients [83].

Administration of AS2 increased SOD and CAT activity in erythrocytes on day 28 (Figure 6a,b). Similar to our findings, supplementation with astaxanthin (2 mg/kg/day) led to an increase in SOD and CAT activity in the brains of mice, which was also associated with an increase in the level of reduced glutathione in the brain [84]. Also, in another study, administration of astaxanthin at a higher dose (100 mg/kg) increased SOD and CAT activity and decreased the serum level of the marker of oxidative lipid damage, malondialdehyde [85]. In this study, we also found increased GPx activity in erythrocytes on day 28 (Figure 6c) at both doses administered, mainly by AS2. This is in agreement with the results of Zhang et al., who administered astaxanthin at doses of 72 mg/kg and 144 mg/kg to rats with gouty arthritis and found significantly higher GPx activity compared to the untreated group [55]. At the same time, we observed an increase in the total antioxidant capacity of plasma (TEAC) in the group supplemented with the higher dose of astaxanthin (AS2) compared to the untreated group (AA), the MTX-treated group, and the group administered with the lower dose of astaxanthin (AS1) (Figure 6d). The antioxidant effects of astaxanthin were also confirmed by a study on healthy rats supplemented with astaxanthin (1 mg/kg) [86]. Our results show that a higher dose of astaxanthin (AS2) significantly reduced the plasma LPx level, compared to the untreated rat group (AA) and the MTX-treated group (Figure 6e). Similar results were found in retinol-deficient rats, where administration of astaxanthin increased activity of antioxidant enzymes (SOD, CAT), glutathione levels, and decreased LPx levels in plasma, liver homogenate, and liver microsomes [87].

In the pivotal experiment comparing organic (ASTAP) and synthetic astaxanthin (ASYN) monotherapy, both showed similar quantitative effects but differed qualitatively. ASTAP reduced hind paw swelling on days 14 and 28 and MMP-9 levels on day 28. ASYN decreased GGT activity, significantly reduced IL-17A and MMP-9 levels on day 28, and increased reduced CoQ_9_ in the liver. Similarly, Kumar et al. found that oral administration of astaxanthin (50 and 100 mg/kg) exhibited significant anti-arthritic activity via enhancing the nociceptive threshold, reducing paw edema, and improving arthritis scores [85]. Pashkow et al. (2008) also studied astaxanthin’s antioxidative and anti-inflammatory properties. This may provide insights into its therapeutic effects in various conditions we discuss in this manuscript [88]. Our results might also be supported by Ambati et al. (2014), a review that provides comprehensive insights into the biological activities of astaxanthin, supporting its therapeutic efficacy [3]. Last but not least, Fassett and Coombes (2011) found in a small number of clinical studies that no adverse events have been reported, and there is evidence of a reduction in oxidative stress and inflammation biomarkers with astaxanthin administration [89]. This article discusses the anti-inflammatory and antioxidative effects of astaxanthin, which is effective in reducing inflammatory markers like IL-17A and MMP-9, which also supports our results. Similarly, Hussein et al. (2006) highlight the health benefits of astaxanthin, including its effects on inflammation and oxidative stress [40].

For combined therapy, ASYN + MTX was superior to ASTAP + MTX, improving weight loss (day 14), hind paw swelling (days 14 and 21), and arthrogram results (day 14), decreasing IL-17A (day 14) and MMP-9 (day 28) levels, GGT activity, and increasing reduced CoQ_9_ levels. Naguib et al. (2000) studied the antioxidant activities of astaxanthin and related carotenoids. The results showed that astaxanthin had the highest antioxidant activity toward peroxyl radicals. The relative reactivities of Trolox, astaxanthin, alpha-tocopherol, alpha-carotene, lutein, beta-carotene, and lycopene were determined to be 1.0, 1.3, 0.9, 0.5, 0.4, 0.2, and 0.4, respectively [90]. The measured antioxidant activity of astaxanthin could explain its effect on oxidative stress markers like GGT and CoQ_9_.

As a standard for carotenoids, β-carotene (BEKA), a vitamin A precursor with antioxidant properties [91], was selected. However, the effect it had on the disease correction by adjusting the measured parameters was the lowest.

β-cryptoxanthin (KXAN), applied at a relatively low dose of 10 μg/kg, was even more effective than ASTAP and ASYN in monotherapy in some parameters. We observed this effect on day 21 for weight adjustment (Figure 7), on days 21 and 28 for swelling reduction (Figure 8), and on day 28 for adjustment of the plasma MMP-9 level (Figure 12). The ability of KXAN to reduce the protein level of MMP-9 in gastric cancer cells was also described previously [92]. Weight loss is an important biometric parameter for monitoring AA development. Already on day 14, MTX and combined therapies prevented weight loss, as did ASYN in monotherapy. However, a significant effect on day 14 was observed only for the ASYN + MTX combination (Figure 7). This or a similar effect was not reported previously in the context of the combination of synthetic astaxanthin and MTX. However, the impact of the combination of different substances with MTX to ameliorate weight loss seen during AA was demonstrated previously [93,94]. Combination therapy with MTX might be a good therapeutic strategy to prevent weight loss during AA. On day 14, the swelling was reduced statistically significantly by all therapies (MTX, combined, and monotherapy). MTX and the ASYN + MTX combinations were the most effective, followed by ASTAP + MTX, and monotherapies were third in order. We can see a similar pattern for the arthrogram on day 14 (Figure 9). On day 21, the ASYN + MTX combination reduced swelling best, followed by MTX and ASTAP + MTX. KXAN monotherapy was nearly as effective. All of these findings were statistically significant. 

Arthritis scores were also assessed in the pivotal study (Table 1). MTX modified the arthrogram values throughout the experiment, statistically significantly on day 14. Both combination therapies and KXAN monotherapy had similar effects. The therapeutic efficacy decreased in the order of ASYN + MTX, MTX, ASTAP + MTX, and KXAN. The combination of ASYN + MTX on day 14 and KXAN on day 28 was the most effective. 

In the pivotal experiment, KXAN at an oral dose of 10 μg/kg daily showed an anti-arthritic effect reflected by amelioration of hind paw swelling, an arthrogram, and by lowering the MMP-9 level on day 28, as described later. Similarly, in a study by Imada et al. (2016), oral administration of β-cryptoxanthin (0.1–1 mg/kg) to antigen-induced arthritic rats suppressed the loss of glycosaminoglycans in articular cartilage. It inhibited IL-1α-induced aggrecan degradation in porcine articular cartilage explants [95]. In humans with inflammatory polyarthritis, a modest increase in β-cryptoxanthin daily intake was associated with a reduced risk of developing inflammatory disorders such as rheumatoid arthritis [96,97]. 

In the pivotal study, the observations from the previous experiments were confirmed, indicating that GGT activity in the spleen is increased due to the influence of the disease (Figure 10). It was described that various antioxidant and anti-inflammatory active substances can modify GGT activity [35,93,98]. 

Regarding plasmatic IL-17A levels, which were measured in both experiments, while no effect on this parameter was observed on day 28 in the first experiment with the two lower doses of astaxanthin (Figure 3b), the higher dose of both natural and synthetic astaxanthin in the second experiment reduced the level of this monitored parameter (Figure 11). In combination with MTX, a significant effect was already achieved on day 14, while of the monotherapies, only ASYN reduced the concentration of IL-17A. An in vitro study by Venkidasamy et al. found that astaxanthin reduced IL-17A levels in mammary epithelial cells exposed to LPS. The authors found that inhibition of histone deacetylases is the main pathway by which astaxanthin antagonizes LPS-induced inflammatory responses in mammary epithelial cells [99]. Another study showed in the LPS-induced otitis media with effusion (OME) rat model that astaxanthin treatment resulted in suppression of IL-17 in the middle ear mucosa of rats. The mechanism behind this effect of astaxanthin might be explained by suppression of the Notch1/Hes1/mTORC1/S6K1 pathway and regulation of Th17/Tregs [100]. In addition to the aforementioned mechanisms by which astaxanthin can regulate IL-17A concentrations in various inflammatory models, Zhao et al., in a 2021 study of a mouse spinal cord injury model, found that astaxanthin inhibited extracellular signal-regulated kinase (ERK)1/2 phosphorylation, p38 mitogen-activated protein kinase (p38 MAPK), and NF-κB p65 phosphorylation and thus inhibited the inflammatory response [56]. The rat CFA model confirmed the efficacy of astaxanthin at higher doses (25, 50, and 100 mg/kg and above) to reduce limb swelling, TNF-α, CRP, and anti-cyclic citrullinated peptide antibodies. This study was the closest in design to our experiment. It differed not only in the spectrum of monitored parameters but also in the antirheumatic drug used for combined treatment [85].

Due to the complexity of the anti-arthritic effect of astaxanthin in our experiment, we also monitored the level of MMP-9 in the plasma (Figure 12). On day 28, in contrast to MTX, which was not effective, the combinations caused a decline in MMP-9 level, while the ASYN + MTX combination was the most effective, followed by ASTAP, KXAN, ASTAP + MTX, and BEKA. The ability of astaxanthin to regulate MMP-9 could be attributed to the mechanism of inhibiting the activation of the JNK and NF-kB pathways, thus modulating the activity and expression of MMP-9 in different areas of the organism [101,102,103]. Other authors describe the ability of astaxanthin to reduce the expression and activity of MMP-9 in the brain after experimental subarachnoid hemorrhage in rats [104].

## 4. Materials and Methods

### 4.1. Animals and Experimental Model of Adjuvant Arthritis

In this complex study, Lewis male rats were obtained from the Department of Toxicology and Laboratory Animal Breeding, Centre of Experimental Medicine, SAS, Dobrá Voda, Slovak Republic (SK CH 20021). Immediately after the housing of animals, rats were placed in a seven-day quarantine. Animals had unlimited access to a standard diet, tap water ad libitum, and a dark/light regime of 12 h/12 h. Animal housing followed the EU Convention for the Protection of Vertebrate Animals Used for Experimental and Other Purposes. The authorization of the protocol for both experiments (pilot and pivotal) was obtained from the Ethics Committee of the Institute of Experimental Pharmacology and Toxicology, Center of Experimental Medicine SAS in Bratislava, Slovakia (SK UCH 04022), and the State Veterinary and Food Administration of the Slovak Republic, Bratislava (3144/16-221/3). Following Directive 2010/63/EU [105], we have implemented the 3Rs (Replacement, Reduction, and Refinement) principle. The application of the 3Rs is currently also embedded in scientific guidance at the European and international ICH (International Cooperation on Harmonization of Technical Requirements) levels [106].

### 4.2. Adjuvant Arthritis (AA) in Lewis Rats

AA is a well-established model of inflammation [38,107]. It is suitable for studying molecular mechanisms between T cells and their subpopulations [108]. AA was induced in rats with a weight of 160–180 g (6 weeks) by individual intradermal immunization at the base of the tail with a suspension of 0.1 mL of 12 mg/mL, heat-inactivated *Mycobacterium butyricum* powder (Difco Laboratories Inc., Detroit MI, USA) suspended in incomplete Freund’s adjuvant (Thermofisher Scientific Inc., Waltham MA, USA) according to our previous protocol [109,110].

### 4.3. The Design of the Pilot AA Experiment (Monotherapeutic Setting)

The rats were randomized into five experimental groups (Table 2). Groups contained 8–16 animals. Group one was used as a healthy control. The second was the untreated AA group. The remaining three monotherapeutic groups were AA rats treated as given in the study design below.

Astaxanthin (AS) and Methotrexate (MTX) (Ebewe, Unterach am Attersee, Austria were administered perorally (via a gastric tube) throughout the entire experiment (28 days); AS was administered daily in two doses (1 mg/kg and 5 mg/kg of b.w.), diluted in canola oil, and the standard treatment, anti-rheumatic drug MTX, was diluted with tap water and applied two times a week in a dose of 0.3 mg/kg of b.w. 

### 4.4. The Design of the Pivotal AA Experiment (Combinational Therapy with MTX)

The rats were randomized into nine experimental groups (Table 3). Groups contained 8–9 animals. Group one was used as a healthy control. The second was the untreated AA group. The remaining five monotherapeutic groups were AA rats treated as given in the study design below:

The tested substance and MTX were administered perorally (via a gastric tube) throughout the entire experiment (28 days); astaxanthin of natural origin, *Blakeslea trispora* (ASTAP), was administered daily in a dose of 20 mg/kg of b.w.; astaxanthin of synthetic origin (ASYN) was administered daily in a dose of 20 mg/kg of b.w.; β-carotene (BEKA) was administered daily in a dose of 20 mg/kg of b.w.; β-cryptoxanthin (KXAN) was administered daily in a dose of 10 μg/kg of b.w. Every substance was diluted in canola oil before administration. A standard treatment, the anti-rheumatic drug MTX, was diluted with tap water and applied two times a week at a dose of 0.3 mg/kg of b.w. 

### 4.5. The Biological Material Collection

On days 14 and 28, blood was drawn into heparinized tubes from the rat’s retro-orbital plexus using light tiletamine Zoletil^®^ (VIRBAC, Hamilton, New Zealand)/Xylazine (Xylariem^®^, Ecuphar, Sintra, Portugal) anesthesia. On the last experimental day, rats were sacrificed under deep Zoletil^®^/Xylazine, and blood was collected from all animals. Blood samples were centrifuged for 15 min at 4 °C to obtain plasma. The samples were stored at −80 °C.

### 4.6. Evaluation of Experimental AA

On days 14, 21, and 28 after the immunization of the animals, the volume of hind paw joints was assessed. Hind paw volume (HPV) was expressed as the average elevation of the percentage (%) of hind paw volume of each rat, compared to HPV measured on day 1 using a water plethysmometer (UGO BASILE, Comerio-Varese, Italy). The HPV on the selected day was divided by the HPV on day 1 and expressed as a percentage according to the following formula:

([Day n]/[Day 1]) × 100 − 100 = value [%].

In both experiments (pilot and pivotal), the animals’ body weight served as a crucial indicator of overall health and response to the experimental conditions. To ensure precise dosing and monitor potential physiological alterations, the body weight of each animal was measured weekly throughout the duration of the study.

To calculate changes in body weight on specific days, a standardized approach was used. This involved subtracting the initial b.w. on day 1 from the respective b.w. measurement on the specified day (day n). The resulting value, expressed in grams, represented the change in b.w. relative to the baseline measurement.

Mathematically, this calculation was represented as follows:

Change in Body Weight (g) = Body Weight on Day n (g) − Body Weight on Day 1 (g)

The arthritic score (AS) is a quantitative parameter determined by combining measurements from multiple biometric inputs associated with arthritis progression. Firstly, the hind paw volume, expressed in milliliters (mL), serves as an indicator of inflammation and swelling in the hind limbs of the animals. The hind paw volume was assessed using established plethysmometry, which measures fluid displacement in a chamber caused by the immersion of the paw. The maximum possible score for hind paw volume is 8 points, reflecting the severity of swelling.

Secondly, the forelimb paw diameter was measured in millimeters (mm). This parameter evaluates the diameter of the forelimb paws and provides additional information on joint inflammation and swelling. The maximum score for forelimb paw diameter is 5 points.

Combining these two inputs—hind paw volume and forelimb paw diameter—we calculated the arthritic score for each animal. This approach allows for a more nuanced assessment of arthritis severity, capturing both local and systemic manifestations of the disease. This well-established parameter has served many times before [35,111,112] as a valuable tool for quantifying disease progression and evaluating the effectiveness of therapeutic interventions in preclinical studies of arthritis.

### 4.7. Experimental Molecules Used in the Study

In the pilot experiment, astaxanthin from natural origin (*Blakeslea trispora*), C_40_H_52_O_4,_ Sigma-Aldrich, St. Louis, MO, USA, was used at doses of 1 mg/kg and 5 mg/kg per b.w.

In the pivotal experiment, astaxanthin from natural origin (*Blakeslea trispora*), C_40_H_52_O_4,_ Sigma-Aldrich, was used in doses of 20 mg/kg of b.w.; astaxanthin from synthetic origin, United States Pharmacopeia (USP) reference standard, was used in doses of 20 mg/kg of b.w.; β-carotene, C_40_H_56_, bought from Sigma-Aldrich, was used in the doses of 20 mg/kg of b.w.; β-cryptoxanthin, C_40_H_56_O, bought from Sigma-Aldrich, was used in the doses of 10 μg/kg of b.w.

### 4.8. Evaluation of AA by Assessment of the Key Markers of Inflammation in Plasma

According to our previous protocol [93,98], inflammatory changes in plasma from AA animals were evaluated on days 14 and 28 of the experiment for IL-17A and MMP-9. Heparinized blood was collected for further plasma evaluation and was determined by the commercial ELISA kits: IL-17A by Immunology Consultant Laboratories, Inc., Portland, OR, USA, and MMP-9 by R&D Systems, Minneapolis, MN, USA. All plasma samples were stored at −80 °C until immunological ELISA analysis.

### 4.9. The Activity of Gamma-Glutamyl Transferase in the Spleen Tissue

As in our earlier experiment [93], we utilized the method of Orlowski and Meister (1970) [113], modified by Ondrejickova et al. (1993) [114], to evaluate the activity of gamma-glutamyl transferase (GGT) on day 28 in the spleen tissue homogenates. The tissue was homogenized for 1 min at 0 °C using Ultra Turax TP 18/10 (Janke and Kunkel, Cologne, Germany) in a phosphate buffer (pH 8.1, 2.6 mM NaH_2_PO_4_, 50 mM Na_2_HPO_4_, 68 mM NaCl, 15 mM EDTA). The biochemical substrates were subsequently diluted to the final concentrations of 2.5 mM and 12.6 mM, respectively, in isopropyl alcohol (65%). The biochemical substrates were 44 mM of methionine and 8.7 mM of l-glutamyl-*p*-nitroanilide. The samples were incubated for an hour at 37 °C before the reaction was stopped by adding 2.3 mL of cold methanol. The tubes were centrifuged for 20 min (Eppendorf centrifuge) at a centrifugal force of 1957× *g*. The absorbance of the supernatant (product *p*-nitroaniline) was determined at 406 nm using a spectrophotometer Specord 40 (Analytikjena, Jena, Germany). Solution mixtures with or without a substrate or an acceptor were utilized as blanks. The activity was determined based on an absorbance measurement and a calibration coefficient. The following formula was used:

a = (A/k) × (3.2/0.5) × (1 × 13.813) × (1000/tissue [mg]) × (1/60) × 1000  = [nmol p − nitroaniline/min/g tissue].

Mathematical formula explanatory note:

A—absorbance;
k—coefficient from calibration curve A = k × c;
c—concentration of p-nitroaniline [µg/mL].

### 4.10. Isolation of mRNA, Reverse Transcription, and Quantitative Real-Time PCR for Measurement of mRNA Expression of HO-1, IL-1β, and PAF-AH

Total mRNA isolation from rat livers using RNAzol^®^ RT (MRC Inc., Cincinnati, OH, USA) and qRT-PCR on a QuantStudio™ 3 thermocycler (Applied Biosystems, Thermo Fisher Scientific, Foster City, CA, USA) has already been extensively addressed by Chrastina et al. (2022) [70]. Takara Primescript™ RT Reagent Kit (Takara Bio Inc., Kusatsu, Shiga, Japan) was used to reverse transcribe isolated mRNA into cDNA following the manufacturer’s instructions. Using HOT FIREPol EvaGreen qPCR Mix Plus (Solis BioDyne, Tartu, Estonia) and the primers for HO-1, IL-1β, and PAF-AH, cDNA was amplified. The combination of two endogenous controls, β-actin and malate dehydrogenase 1 (MDH1), was used to evaluate the relative mRNA expression using the Pfaffl calculations.

### 4.11. Determination of Coenzyme Q_9_ (CoQ_9_) in Plasma and Liver Tissue

Concentrations of CoQ_9_ were measured by the HPLC method using the isocrating pump Alpha 10 and the variable wavelength detector Sapphire (both ECOM Ltd., Chrastany u Prahy, Czech Republic) as described previously [68]. CoQ_9_ concentrations (total) (CoQ_9_Tot) in plasma were measured after oxidation with 1,4-benzoquinone (Merck, Darmstadt, Germany), according to Mosca et al., 2002 [115]. Plasma extraction was performed according to Lang et al., 1986 [116], with some modifications. 500 μL of plasma was supplemented with 100 μL 1,4-benzoquinone solution (2 mg/mL double distilled water) and vortexed for 10 s. After 10 min, 2 mL of the mixture of hexane/ethanol (5/2 *v*/*v*, Merck) was added, shaken for 5 min, and centrifuged. The hexane layer was separated, and the extraction procedure was repeated with 1 mL of the extraction mixture. The collected organic layers were evaporated under nitrogen at 50 °C. The residues were taken up in 99.9% ethanol (Merck) and injected into a Separon SGX C18 7 m 3 × 150 mm column (Tessek, Prague, Czech republic). Elution was performed with methanol/acetonitrile/ethanol/(6/2/2, *v*/*v*/*v*). The processing of the liver tissue was similar [116] for assessing the reduced forms of CoQ_9_ (CoQ_9_red). Liver tissue (approx. 100 mg) was homogenized using an Ultra-Turrax in 1 mL of redistilled water with an addition of 50 μL of t-butylhydroxytoluene (BHT, 10 mg/1 mL of 99.9% ethanol). Homogenate was extracted by a hexane/ethanol mixture (5/2, *v*/*v*) with the addition of 1 mL of 0.1 M sodium dodecyl sulfate (SDS). Concentrations of CoQ_9_Tot were detected spectrophotometrically at 275 nm and CoQ at 290 nm using external standards (Sigma-Aldrich, St. Louis, MO, USA) with three-point calibration curves. A reduced coenzyme Q_9_ standard was prepared by reducing CoQ_9_ with sodium dithionite [116]. Data were collected and processed using a CSW 32 chromatographic station (DataApex Ltd., Prague, Czech republic). Concentrations were calculated as follows: in the plasma in μmol/L and in the liver tissue in nmol/g wet weight.

### 4.12. Measuring the Activity of Antioxidant Enzymes (SOD, GPx, and CAT) in Erythrocytes and Markers of Oxidative Stress (TEAC and LPx) in Plasma Samples

#### 4.12.1. Hemolysate Preparation and Determination of Hemoglobin

Washed erythrocytes were diluted 1:3 (*v*/*v*) with ice-cold distilled water and incubated for 15 min. The hemolysate was then stored at −20 °C until further analysis. The hemoglobin concentration in the hemolysate was measured using the Drabkin method [117] and expressed in g/L.

#### 4.12.2. The Activity of Antioxidant Enzymes

Superoxide dismutase (SOD) activity in erythrocyte hemolysates was measured using a commercial kit (Sigma-Aldrich, No. 19160, St. Louis, MO, USA) according to the manufacturer’s instructions. The activity was reported in units per milligram of hemoglobin (U/mg Hb).

Glutathione peroxidase (GPx) activity in erythrocyte hemolysates was determined using the Glutathione Peroxidase Activity Kit (Cayman Chemical, No. 703102, Ann Arbor, MI, USA) according to the manufacturer’s instructions. The activity was expressed in micromoles of substrate converted per second per milligram of hemoglobin (µkat/mg Hb).

Catalase (CAT) activity in erythrocyte hemolysates was measured based on the method described by Bergmeyer [118]. This method monitors the decrease in absorbance of hydrogen peroxide at 240 nm over time. Catalase activity was expressed in micromoles of substrate converted per second per gram of hemoglobin (µkat/g Hb).

#### 4.12.3. Markers of Oxidative Stress

The concentration of lipoperoxides in serum was determined according to El-Saadani et al. [119]. This method utilizes the ability of peroxides to oxidize iodide (I^−^) to iodine (I_2_). The formed iodine reacts with excess iodide to form triiodide (I_3_^−^), which has an absorption maximum at 365 nm. The lipoperoxide concentration was expressed in nmol/mL.

The Trolox equivalent antioxidant capacity (TEAC) assay measured the ability of antioxidants in serum to scavenge the stable radical cation ABTS^+^ (2,2′-azinobis(3-ethylbenzothiazoline-6-sulfonic acid)). This assay employs a blue-green chromophore with an absorbance peak at 734 nm, which is decolorized by both lipophilic and hydrophilic antioxidants present in the sample. The antioxidant activity was compared to Trolox, a synthetic vitamin E analogue [120] and expressed as mmol Trolox equivalents per litre (mmol/L).

### 4.13. Statistical Evaluation of the Experimental Results

The arithmetic mean (average value) and ± standard error of the mean (SEM) were applied to express the experimental results. The statistically significant differences between the experimental groups were determined using the GraphPad InStat3 (GraphPad Software, Boston MA, USA) program. A one-way ANOVA with a Tukey–Kramer post hoc test was used to evaluate any significant differences between the control animals (HC), untreated animals (AA), and the treatment groups of animals (MTX, AS1, AS2, ASTAP, ASYN, BEKA, KXAN, ASYN + M, and ASTAP + M). AA was compared to HC, all treated groups were compared to AA, and ASYN + M and ASTAP + M, respectively, were compared to MTX. The post hoc test (Tukey–Kramer) was used when significant variations existed between the groups. Following the post hoc screening, the following degrees of significance were established: not significant (*p* > 0.05), significant (*p* ≤ 0.05), very significant (*p* ≤ 0.01), and highly significant (*p* ≤ 0.001). The legend beneath each table and graphic provides information about the specific symbol of significance.

### 4.14. Limitations of the Study

Only part of the complex pathology of a disease like RA may be demonstrated using animal models. Understanding that human disease is a condition that can arise in individuals due to a variety of environmental exposures and immunogenetic regulation makes this even more difficult. It may not be reasonable to expect inbred animals exposed to a particular arthritogenic stimulus to represent the complicated modality of RA. It is up to the investigator to determine if the model represents a relevant element of human pathology and then draw suitable conclusions. Animal models are effective because they do not include confounding variables that could eventually account for the limited effectiveness of therapy when treating the human condition. The “non-specific” character of the immune activation leading to joint illness may be another advantageous aspect of adjuvant arthritis [121].

Similar to RA’s uncertain etiology, the mineral oil’s function and the mycobacterial components of complete Freund’s adjuvant (CFA) in inducing joint illness are still unknown. The adjuvant activation process can lead to systemic overstimulation of macrophages, which can result in the rise of autoimmune T and B cell populations. These immune cells may then result in joint infiltration and the generation of various autoantibodies against heat shock protein (Hsp) and cartilage antigens. Furthermore, cytokines produced from macrophages directly affect chondrocytes and synovial cells, causing atypical matrix component synthesis, aberrant expression of adhesion molecules and MHC class II antigens, and synovial hypertrophy. The quick disease onset (16–19 days), or even sooner, as presented in this paper (around day 10), which is unlikely typical of the gradual onset and chronic nature of the rheumatoid disease, may be another drawback of adjuvant arthritis in simulating RA. However, in the pharmaceutical sector, where financial limitations might prevail over scientific model accuracy, early illness onset is seen as a significant benefit [122].

The number of animals is also a limitation, as are the limitations regarding the 3Rs, the pharmacokinetics of experimental substances, and pharmacokinetic interactions that could explain some phenomena. Last but not least, the fact that we did not analyze the mechanisms of action at the molecular level is also a limitation.

## 5. Conclusions

To conclude, in the first experiment, despite the low doses of astaxanthin (1 and 5 mg/kg), the higher dose (5 mg/kg) (AS2) was found to reduce body weight loss on day 14 and moderate the increase in hind paw volume on day 14. AS2 also showed a significant effect on reducing the activity of GGT in the spleen (whose increased activity contributes to inflammatory processes) and reducing plasma MMP-9 levels at day 28. In addition, AS2 decreased the relative mRNA expression of HO-1 in the liver compared with the lower dose of AS1. Further, AS2 increased the activity of the antioxidant enzymes SOD, GPx, and CAT in erythrocytes. It also increased the antioxidant capacity (TEAC) and decreased the plasma LPx concentration compared to AA. 

In the second experiment, the astaxanthin dose was increased to 20 mg/kg, and its effect was compared with other representatives of carotenoids—β-carotene (BEKA) and β-cryptoxanthin (KXAN). The different sources of astaxanthin (synthetic and organic) were compared. The dose dependence was confirmed; the highest dose of astaxanthin (20 mg/kg) had an effect on individual parameters even in monotherapy.

The differences between ASTAP and ASYN are described in the literature. ASTAP is evaluated in superlatives, and its several times higher antioxidant activity is described; it is recommended as a source of astaxanthin for humans, while ASYN is recommended for administration to animals and humans only in low doses. If we compare ASTAP and ASYN in monotherapy in the presented study, their effect was approximately the same quantitatively. However, they qualitatively differed in efficacy because each affected different parameters. Based on our observations, therefore, a “better” source of astaxanthin cannot be easily determined.

The results of the second experiment showed that β-cryptoxanthin (KXAN), which was applied at a relatively low dose of 10 μg/kg, was even more effective than ASTAP and ASYN in monotherapy in some parameters.

For combined therapy, the effect of ASYN + MTX was clearly better compared to ASTAP + MTX.

The results of the presented work brought original findings in the field of the use of substances of natural origin from the group of carotenoids as possible supplements to the classical strategy of RA therapy. 

These findings, especially the anti-arthritic effect of KXAN and ASYN + MTX, can be the basis for further preclinical studies and will be the subject of further research.

## Figures and Tables

**Table 1:** The table shows the arthrogram in points given to experimental animals measured on days zero (beginning of the experiment), 7, 14, 21, and 28 of the pivotal experiment.

Group	Day 0	Day 7	Day 14	Day 21	Day 28
HC	8 ± 0	8 ± 0	9.13 ± 0.35	9.75 ± 0.16	10 ± 0
AA	7.88 ± 0	8 ± 0	11.62 ± 0.75 *	13 ± 0.71 *	13.25 ± 0.59
MTX	8 ± 0	8 ± 0	8.78 ± 0.36 ++	10.89 ± 0.48	11.56 ± 0.53
ASTAP + MTX	8 ± 0	8 ± 0	9.67 ± 0.47	11.44 ± 0.8	12.22 ± 0.52
ASYN + MTX	7.78 ± 0.22	8.00 ± 0	8.44 ± 0.34 ++	10.22 ± 0.64	11.78 ± 0.72
ASTAP	8.00 ± 0	8.22 ± 0.15	10.33 ± 0.53	13.38 ± 0.71	12.13 ± 0.48
ASYN	8.00 ± 0	8.13 ± 8.13	10.88 ± 0.48	14 ± 0.50	12.22 ± 1.62
BEKA	8.00 ± 0	8 ± 0	10.88 ± 0.52	13.75 ± 0.53	13 ± 0.46
KXAN	8.00 ± 0	8.11 ± 0.11	10.56 ± 0.58	11.89 ± 0.87	11.78 ± 0.83

HC—healthy control group, AA—group of untreated controls with adjuvant arthritis (AA); MTX—rats with AA treated with methotrexate (MTX) at a dose of 0.3 mg/kg twice weekly; ASTAP—group of rats with AA receiving astaxanthin of natural origin in the oral dose of 20 mg/kg daily; ASTAP + MTX—group of rats with AA receiving astaxanthin of natural origin in the oral dose of 20 mg/kg daily and in a combination with MTX at a dose of 0.3 mg/kg twice weekly; ASYN—group of rats with AA receiving synthetic astaxanthin in the oral dose of 20 mg/kg daily; ASYN + MTX—group of rats with AA receiving synthetic astaxanthin in the oral dose of 20 mg/kg daily and in combination with MTX at a dose of 0.3 mg/kg twice weekly; BEKA—group of rats with AA receiving β-carotene in the oral dose of 20 mg/kg daily; KXAN—group of rats with AA receiving β-cryptoxanthin in the oral dose of 10 μg/kg daily. Data are expressed as mean ± SEM. Statistical significance was evaluated using ANOVA for independent variables: * *p* < 0.05 vs. HC group, ++ *p* < 0.01 vs. AA group.

**Table 2:** The experimental animals were divided into five groups for the pilot experiment.

Group	Treatment
HC (healthy control)	Vehiculum—canola oil
AA (untreated AA animals)	Vehiculum—canola oil
MTX (AA animals treated with MTX)	MTX 0.3 mg/kg, twice a week
AS1 (AA animals treated with astaxanthin)	AS (source *Blakeslea trispora*), 1 mg/kg, daily
AS2 (AA animals treated with astaxanthin)	AS (source *Blakeslea trispora*), 5 mg/kg, daily

HC—healthy control; AA—adjuvant arthritis; AS—astaxanthin; MTX—methotrexate.

**Table 3:** The experimental animals were divided into nine groups for the pivotal experiment.

Group	Treatment
HC (healthy control)	Vehiculum—canola oil
AA (untreated AA animals)	Vehiculum—canola oil
MTX (AA animals treated with MTX)	MTX 0.3 mg/kg, twice a week
ASTAP + MTX (AA animals treated with AS-natural and MTX)	AS (source *Blakeslea trispora*), 20 mg/kg daily + MTX 0.3 mg/kg twice a week
ASYN + MTX (AA animals treated with AS-synthetic and MTX)	AS (synthetic) 20 mg/kg daily + MTX, 0.3 mg/kg twice a week
ASTAP (AA animals treated with AS-natural and MTX)	AS (source: *Blakeslea trispora*), 20 mg/kg daily
ASYN (AA animals treated with AS-synthetic and MTX)	AS (synthetic), 20 mg/kg daily
BEKA (AA animals treated with β-carotene)	β-carotene, 20 mg/kg daily
KXAN (AA animals treated with β-cryptoxanthin)	β-cryptoxanthin, 10 μg/kg daily

HC—healthy control; AA—adjuvant arthritis; AS—astaxanthin; MTX—methotrexate.

## Data Availability

The raw data supporting reported results can be found in the following location: https://doi.org/10.6084/m9.figshare.26139877.v1 (accessed on 1 July 2024).
